# Metallic Co Nanoarray Catalyzes Selective NH_3_ Production from Electrochemical Nitrate Reduction at Current Densities Exceeding 2 A cm^−2^

**DOI:** 10.1002/advs.202004523

**Published:** 2021-02-01

**Authors:** Xiaohui Deng, Yongpeng Yang, Lei Wang, Xian‐Zhu Fu, Jing‐Li Luo

**Affiliations:** ^1^ Shenzhen Key Laboratory of Polymer Science and Technology Guangdong Research Center for Interfacial Engineering of Functional Materials College of Materials Science and Engineering Shenzhen University Shenzhen 518061 China; ^2^ Henan Institute of Advanced Technology Zhengzhou University Zhengzhou 450001 China

**Keywords:** ammonia production, density function theory calculation, electrochemical nitrate reduction, in situ reduction, nanoarrays

## Abstract

Electrochemical nitrate reduction (NITRR) offers a promising alternative toward nitrogen recycling and ammonia production under ambient conditions, for which highly active and selective electrocatalyst is desired. In this study, metallic cobalt nanoarrays as facilely prepared from the electrochemical reduction of Co(OH)_2_ nanoarrays (NAs) are demonstrated to exhibit unprecedented NH_3_ producing capability from catalyzing NITRR. Benefitting from the high intrinsic activity of Co^0^, intimate contact between active species and conductive substrate and the nanostructure which exposes large number of active sites, the Co‐NAs electrode exhibits current density of −2.2 A cm^−2^ and NH_3_ production rate of 10.4 mmol h^−1^ cm^−2^ at −0.24 V versus RHE under alkaline condition and significantly surpasses reported counterparts. Moreover, the close‐to‐unity (≥96%) Faradaic efficiency (FE) toward NH_3_ is achieved over wide application range (potential, NO_3_^−^ concentration and pH). Density function theory calculation reveals the optimized adsorption energy of NITRR intermediates on Co surface over Co(OH)_2_. Furthermore, it is proposed that despite the sluggish kinetics of Volmer step (H_2_O → *H + *OH) which provides protons in conventional hydrogenation mechanism, the proton‐supplying water dissociation process on Co surface is drastically facilitated following a concerted water dissociation–hydrogenation pathway.

## Graphical abstract

Metallic Co‐NAs that are electrochemically reduced from Co(OH)_2_‐NAs exhibit record‐high electrocatalytic activity and close‐to‐unity Faradaic efficiency toward ammonia production from nitrate reduction. DFT calculations reveal that different from the common hydrogenation pathway, fast water dissociation assisted by intermediates with unsaturated N atoms and their concurrent hydrogenation contribute to the superior NITRR activity.

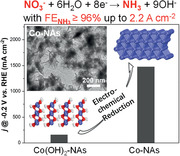

## 1 Introduction

Ammonia (NH_3_) plays significant roles in multiple aspects of human livings and is one of the most common industrial chemicals. Viewed by many as the “greatest” scientific invention of the 20th century, “Haber–Bosch” process paves a way toward the mass production of ammonia by reacting nitrogen with hydrogen under high temperature and pressure.^[^ ^1^ ^]^ Although it still dominates the ammonia production industry, novel ammonia production methods are being actively pursued as the harsh operation condition of “Haber–Bosch” process leads to 2% of the global annual energy consumption and more than 0.5% of the global CO_2_ emission (2015).^[^ ^2^ ^]^ In stark comparison, photo/electrochemical N_2_ reduction reaction (NRR) stands out as a promising alternative.^[^ ^3^ ^]^ This approach, which can be operated under ambient conditions, extract protons from H_2_O molecules and is free of emission.^[^ ^4^ ^]^ Despite such advantages, the inert character of N_2_ molecule and competing hydrogen evolution reaction (HER) often leads to limited FE and extremely low ammonia production rate, making it unsuitable for mass production up to now.^[^ ^5^ ^]^

On the other hand, nitrate (NO_3_^−^) anion is regarded as a N‐containing pollutant in nature.^[^ ^6^ ^]^ Factors such as extensive usage of artificial fertilizers, degradation of household/human waste, combustion of fossil fuels, and industrial activities undesirably result in the accumulation of NO_3_^−^ in the underground water, surface water, animals, and plants. The intake of NO_3_^−^ in human body can potentially lead to diseases such as non‐Hodgkin's lymphoma and methemoglobinemia.^[^ ^7^ ^]^

With the aim to tackle this problem and recycle the fixed nitrogen, electrochemical nitrate reduction (NITRR, NO_3_^−^ + 9H^+^ + 8e^−^ → NH_3_ + 3H_2_O) serves as a “two birds‐one stone” approach as NO_3_^−^ can be potentially converted to ammonia.^[^ ^8^ ^]^ Like NRR, the reaction is performed under ambient conditions and no additional reductant or proton source (other than H_2_O) is required. Examples of nitrate recycling process to produce ammonia haven been outlined in literature.^[^ ^8^, ^9^ ^]^ As the reaction involves multiple intermediates and proceeds through the transfer of 9 protons and 8 electrons, high FE toward NH_3_/NH_4_^+^ is highly desired.^[^ ^10^ ^]^ So far, Ru‐ and Cu‐based electrocatalysts are reported as the most active and selective candidates. A strain‐tuned Ru/O‐doped‐Ru core/shell nanocluster was reported for catalyzing NITRR with an FE close to 100% for NH_3_.^[^ ^11^ ^]^ This electrocatalyst remains highly selective until −0.2 V versus RHE in 1 m KOH with the current density of 130 mA cm^−2^ and a record‐high NH_3_‐synthesizing rate. The intermediate adsorption energies were also optimized on copper‐nickel alloy to enhance the performance and selectivity toward NH_3_.^[^ ^12^ ^]^ Recently, Cu/Cu_2_O heterostructure and oxygen‐deficient TiO_2_ were reported to be able to catalyze NITRR with FE (toward NH_3_) up to 80% under neutral conditions.^[^ ^8a,8b^ ^]^ Although decent progress has been made, the catalyst library remains underexplored and the achieved current density toward NITRR remain lower than 200 mA cm^−2^ in most cases, limiting its practical application in NH_3_ production. Furthermore, the interplay between HER and NITRR on the catalyst surface cannot be neglected as the water‐dissociation Volmer step (H_2_O → *H + *OH)—one of the elementary steps in HER—is crucial in providing protons for hydrogenation of NITRR intermediates. However, robust HER electrocatalyst with fast water‐dissociation kinetics would undesirably favor HER over NITRR as the latter often occurs below the thermodynamic potential required for HER. In this case, the FE shifts to H_2_ production under large current densities at more negative potentials, limiting the current efficiency and NH_3_ production rate. Therefore, in order to achieve NH_3_ production from NITRR with high FE, electrocatalysts that operate through novel mechanistic pathways might overcome this limitation. For instance, aforementioned strain‐tuned Ru/O‐doped‐Ru core/shell nanocluster catalyzes NITRR with a hydrogen‐radical involved pathway by suppressing H‐H dimerization during water splitting.^[^ ^11^ ^]^

Herein, we report HER‐inert monometallic Co nanoarrays can be potentially applied in N recovery from NITRR with unprecedented NH_3_ production capability under both alkaline and neutral conditions. The facilely‐accessible metallic Co nanoarrays (Co‐NAs), which are obtained from the electrochemical reduction of Co(OH)_2_‐NAs, consist of abundant nanosheets and are rich in surface active sites. Benefitting from the outstanding intrinsic NITRR activity of in situ generated Co^0^ and large number of active sites, Co‐NAs show close‐to‐unity FE toward NH_3_ production in a wide potential range (0.11 to −0.24 V vs RHE) with the current densities up to −2.2 A cm^−2^ in 1 m KOH containing 0.1 m NO_3_^−^. The NITRR performance and FE are further maintained during long‐term electrolysis (10 h) at −500 mA cm^−2^. To gain theoretical insights and rationalize the superior NITRR activity of Co‐NAs, density functional theory (DFT) calculations are performed to probe the origin of high NITRR activity and a mechanism which involves the concurrent dissociation of *H_2_O and hydrogenation of key reaction intermediates are proposed.

## 2 Results and Discussion

**Figure 1:**
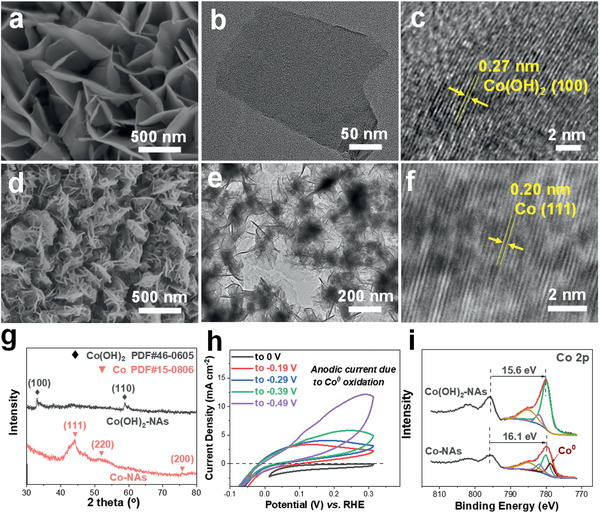
SEM, TEM, and high‐resolution TEM images of a–c) Co(OH)_2_ and d–f) Co‐NAs on carbon cloth. g) XRD patterns of Co(OH)_2_ and Co‐NAs. h) Cyclic voltammetry curves of Co(OH)_2_‐NAs with various potential scanning range (from 0.31 V to 0, −0.19, −0.29, −0.39, −0.49 V vs RHE) in 1 m KOH and i) Co 2p XPS spectra of Co(OH)_2_ and Co‐NAs.

Co(OH)_2_‐NAs are first prepared on carbon cloth (CC) by electrodeposition. Scanning electron microscopy (SEM) images (**Figure** **1**a, Figure S1a, Supporting Information) show that the electrode exhibits 3D open‐pore structure which are constructed by the cross‐linked nanosheets. It is further investigated by transmission electron microscopy (TEM, Figure 1b,c) and the clear lattice fringes with a plane distance of 0.27 nm corresponds well to the (100) plane of *α*‐Co(OH)_2_.^[^ ^13^ ^]^ The X‐ray diffraction (XRD) pattern of Co(OH)_2_‐NAs is shown in Figure 1g and the characteristic reflections at 33.0^o^ and 58.9° can be assigned to the (101) and (110) planes of *α*‐Co(OH)_2_ phase.^[^ ^14^ ^]^

During the initial assessment of NITRR activity, as‐prepared Co(OH)_2_‐NAs show negligible cathodic current from 0.15 to −0.1 V versus RHE, indicating its low intrinsic NITRR activity. Interestingly, the cathodic currents gradually increase from −75 to −465 mA cm^−2^ in 200 s when chronoamperometry measurement is conducted at −0.19 V versus RHE (Figure S2, Supporting Information). Similar trend is observed when applied potential is more negatively shifted. Since Co(OH)_2_ exhibits low activity toward HER under such potential and no gas bubbles can be observed from the electrode surface, it is therefore speculated that NITRR contributes to the significant change of cathodic current. To gain more insights into the “activation” behavior, the variation of the chemical states of Co is first probed by cyclic voltammetry (CV) measurements in 1 m KOH without the presence of nitrates—this way the current contribution from NITRR can be excluded. As presented in Figure 1h, capacitive currents can merely be observed when the potential is scanned till 0 V versus RHE, suggesting electrodeposited Co(OH)_2_ remain stable within this range. By shifting the vertex potential to −0.19 V, an obvious anodic peak with an onset potential of −0.02 V emerges from the anodic scan and this can be assigned to the oxidation of metallic Co^0^.^[^ ^15^ ^]^ Moreover, the area of Co^0^ oxidation peak increases with the vertex potential being more negatively shifted, which is reasonable as higher contents of Co^2+^ would be electrochemically reduced accordingly.

Electrochemical reduction has been recently demonstrated as a facile protocol to prepare metallic electrocatalyst from MOF precursors.^[^ ^8^, ^16^ ^]^ However, the fabrication of first‐row transition metal electrodes is rarely reported due to their limited catalytic applications. Inspired by the enhanced NITRR activity exhibited by in situ generated Co^0^, Co(OH)_2_‐NAs are then taken as the precursor and electrochemically reduced at −0.49 V versus RHE in 1 m KOH. The obtained electrode is thoroughly characterized to reveal the change of physical properties. The SEM images presented in Figure 1d and Figure S1b, Supporting Information, show that the original Co(OH)_2_‐NAs evolve into homogeneous nanobundles consisting of multiple nanosheets with domain size below 300 nm. The TEM images confirm the ultra‐thin feature of the nanosheet (Figure 1e) and lattice spacings of 0.20 nm can be assigned to Co (111) facets (Figure 1f). It should be noted that the elemental mapping of Co‐NAs (Figure S3, Supporting Information) shows the presence of O in significantly lower content comparing with Co(OH)_2_ after electrochemical reduction. The remaining O can be attributed to the surface oxidation due to exposure of Co‐NAs to electrolyte and ambient oxygen once the electrical bias is ceased. The variation of crystal structure is further investigated by XRD. As presented in Figure 1g, the characteristic reflections previously assigned to Co(OH)_2_ disappeared and new reflections at 44.2°, 51.5°, and 75.8° emerged. Further peak position assignments match well with that of metallic Co^0^ (PDF#15‐0806), suggesting the phase transformation induced by electrochemical reduction. X‐ray photon spectroscopy (XPS) is then employed to provide more insights on the chemical states of Co. As plotted in Figure 1h, the Co 2p spectrum of as‐prepared Co(OH)_2_ exhibits dominant peak at 780.2 eV, being in good agreement with literature value.^[^ ^14^, ^17^ ^]^ In comparison, a new peak at 778.6 eV arises after the electrochemical reduction and can be assigned to Co^0^ in Co‐NAs.^[^ ^18^ ^]^ The dominant peak at ≈780 eV can still be observed due to the readily surface oxidation of Co‐NAs. In addition, the spin‐orbit splitting value of Co‐NAs is 16.1 eV in comparison to 15.6 eV of Co(OH)_2_‐NAs, indicating the decrease of Co valence states.^[^ ^14^, ^19^ ^]^

**Figure 2:**
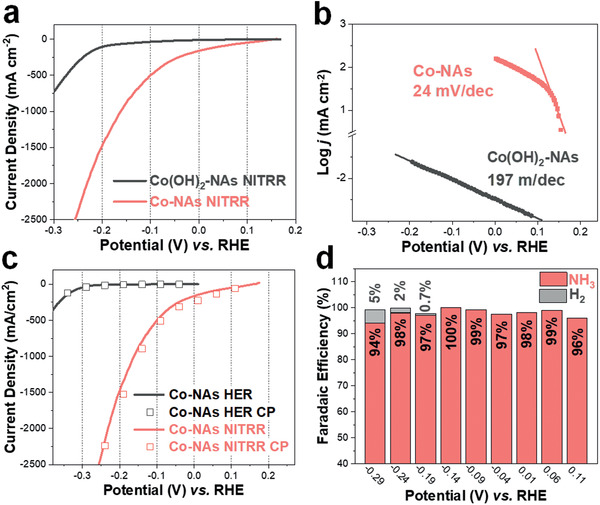
Electrochemical performance of NH_3_ production from nitrate reduction reaction (NITRR). a–b) NITRR LSV curves and derived Tafel plots of Co‐NAs and Co(OH)_2_‐NAs. c) LSV comparison of HER and NITRR catalyzed by Co‐NAs. Squares represent the current densities obtained from chronoamperometric (CA) measurements. d) Potential‐dependent Faradaic efficiency of Co‐NAs toward NH_3_ and H_2_.

The presented characterization results evidence the successful fabrication of nanostructured Co^0^ electrode from electrochemical reduction of Co(OH)_2_‐NAs. The electrocatalytic performance toward NITRR is then systematically investigated in 1 m KOH electrolyte containing 0.1 m of NO_3_^−^. As shown in the LSV curves in **Figure** **2**a, the Co‐NAs exhibit significantly enhanced NITRR activity comparing with pristine Co(OH)_2_. The onset potential is positively shifted to 0.16 V versus RHE and the Tafel plots derived from the LSVs are depicted in Figure 2b. It can be read that Co‐NAs exhibit an ultra‐low Tafel slope of 24 mV dec^−1^. In comparison, Co(OH)_2_‐NAs shows a Tafel slope of 197 mV dec^−1^, reflecting the sluggish NITRR kinetics. It is worth to mention that the sharp increase of NITRR current for Co(OH)_2_‐NAs after −0.2 V is attributed to the in situ generation of highly active Co^0^ species during the linear scan.

We next compare the electrocatalytic activity of Co‐NAs toward HER and NITRR. The HER performance is evaluated in the absence of NO_3_^−^ and the LSV curve plotted in Figure 2c suggests the poor HER activity, reaching −10 and −100 mA cm^−2^ at −0.26 and −0.33 V versus RHE, respectively. In comparison, current densities of −100, −500, and −1000 mA cm^−2^ are reached at 0.04, −0.1, and −0.16 V versus RHE in the presence of NO_3_^−^, demonstrating the superior catalytic activity of Co‐NAs toward NITRR. A series of controlled‐potential chronoamperometric (CA) measurements are further conducted and the steady‐state currents collected from each potential step are plotted as squares (detailed current‐time profiles of HER and NITRR shown in Figures S4 and S5, Supporting Information). As can be observed, the steady‐state currents correlate well with the values collected from the LSVs in both cases. Since HER is considered as the competing side‐reaction, poor HER performance shown by Co‐NAs can greatly benefit the FE toward NITRR. The impact of potential applied during the electrochemical reduction process on the NITRR activity is also investigated. Despite the similar morphologies as revealed by SEM (Figure S6, Supporting Information), it is found that with a reduction time of 300 s, steady state of the electrode is not reached at potentials more positive than −0.39 V versus RHE. The gradually increasing currents in the cases of −0.19 and −0.29 V indicates the generation of new active sites from Co(OH)_2_ reduction (Figure S7, Supporting Information).

**Figure 3:**
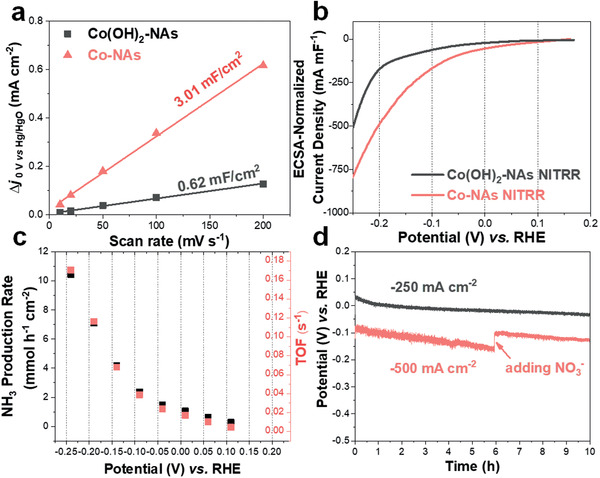
a) Calculated double layer capacitance of Co(OH)_2_‐NAs and Co‐NAs. b) Double layer capacitance normalized‐LSVs of Co(OH)_2_‐NAs and Co‐NAs toward NITRR. c) Calculated NH_3_ production rate and TOF from NITRR catalyzed by Co‐NAs by assuming all Co atoms are catalytically active. d) Chronopotentiometry test of Co‐NAs at the current density of −250 and −500 mA cm^−2^ over 10 h.

With the high NITRR activity of Co‐NAs in hand, the selectivity toward NH_3_ production is then investigated. Standard solutions with known NH_3_ concentrations are first measured using colorimetric method and the calibration curve is obtained with good linearity (Figure S8a, Supporting Information).^[^ ^8a^ ^]^ The electrolyte after NITRR at fixed potentials is then diluted and the NH_3_ concentration is calculated based on the measured absorbance accordingly. Impressively, the FE toward NH_3_ production is above 96% from 0.11 to −0.24 V versus RHE (Figure 2d). The original UV–vis adsorption data of the diluted electrolyte collected at 0.06, −0.09, and −0.24 V and the fitting results are also plotted in Figure S8, Supporting Information, demonstrating the reliability of this method. H_2_, as the only detected side‐product from GC, is present in small quantity at potentials more negative than −0.19 V versus RHE. The concentration of NH_3_ is further quantified by ^1^H NMR with maleic acid as internal standards and similar FE proves the accuracy of colorimetric method (Figure S9, Supporting Information). In order to probe the impact of electrochemical‐reduction protocol on the surface active sites, the capacitance currents at various scan rates are measured and the calculated electrochemically active surface area of the electrode undergoes a fivefold increase after the reduction process, with Co‐NAs exhibiting a normalized capacitance of 3.01 mF cm^−2^ over 0.62 mF cm^−2^ for Co(OH)_2_‐NAs (Figure S10, Supporting Information). To compare the intrinsic activity of Co(OH)_2_‐NAs against Co‐NAs, the capacitance‐normalized LSVs are further plotted in **Figure** **3**b and it can be observed that Co^0^ exhibits significantly enhanced activity. Based on the LSV and determined FE, the geometric surface area‐normalized NH_3_ production rates (${v_{\mathrm{NH}}}_{{}_{2}}$) and turnover frequency (TOF) are plotted against applied potential in Figure 3c. As can be read, the Co‐NAs produce NH_3_ with a rate of 4.16 mmol h^−1^ cm^−2^ at −0.14 V versus RHE. It further reaches 10.4 mmol h^−1^ cm^−2^ at −0.24 V versus RHE, corresponding to a TOF with a lower bound of 0.17 s^−1^ by assuming all the Co atoms on the electrode is catalytically active. It is worth noting that Co‐NAs significantly outperforms recently‐reported strained Ru nanoclusters, NiCu alloy and other counterparts (Table S1, Supporting Information).^[^ ^8^, ^9^, ^11^, ^12^, ^20^ ^]^ We attribute the superior NITRR activity of Co‐NAs to the following factors. First, obtained Co^0^ as active sites exhibit high intrinsic activity and selectivity toward NH_3_ production from NITRR as has been demonstrated by the ultra‐low Tafel slope and close‐to‐unity FE. Second, the electrode preparation protocol ensures the intimate contact between the nanoarrays and conductive substrate; the reduction of Co(OH)_2_ to metallic Co with higher conductivity can further enhance the transfer of charge carriers to the surface active sites.^[^ ^21^ ^]^ Last but not least, the unique nanostructure of Co‐NAs is able to expose large number of active sites. Moreover, long‐term NITRR experiments are performed to evaluate the catalytic/structural stability of Co‐NAs under high NH_3_ production rates and the potentials required to reach the current densities of 250 and 500 mA cm^−2^ are monitored. As can be observed from Figure 3d, Co‐NAs show outstanding NITRR stability as slight decrease of applied potential is observed over a period of 10 h. The more severe change in the case of 500 mA cm^−2^ is attributed to the faster depletion of NO_3_^−^ as the potential shifts immediately upon the recovery of NO_3_^−^ concentration. Moreover, the structure degradation of Co‐NAs is observed to be mild from the SEM images (Figure S11, Supporting Information) and the FE toward NH_3_ production is determined to be 98% at the end of electrolysis, further demonstrating the excepitional stability of Co‐NAs.

**Figure 4:**
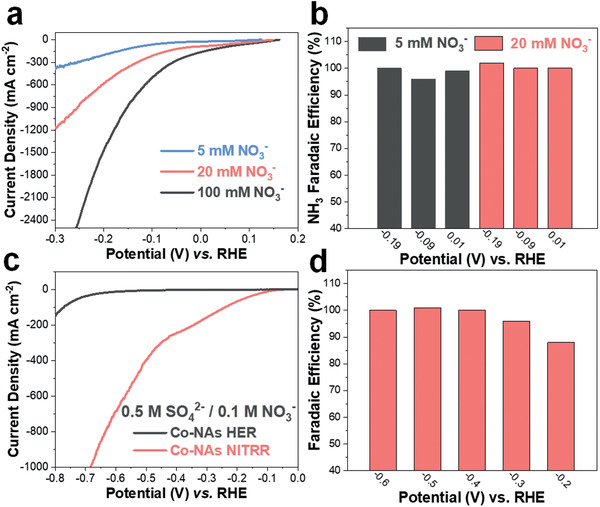
a) NITRR LSV curves of Co‐NAs and b) Faradaic efficiency toward NH_3_ production in 1 m KOH containing 5 and 20 mm NO_3_^−^. c) LSV curve comparison of HER/NITRR and d) Faradaic efficiency toward NH_3_ production from −0.2 to −0.6 V in 0.5 m K_2_SO_4_ containing 100 mm NO_3_^−^.

The applicability of Co‐NAs toward NH_3_ production from NITRR is further studied by varying the NO_3_^−^ concentration and pH of the electrolyte. **Figure** **4**a presents the LSVs collected from 1 m KOH containing 5 and 20 mm NO_3_^−^. As can be expected, the current densities decrease with lower concentration of NO_3_^−^. Importantly, negligible impact on the product selectivity is observed (Figure 4b) as FE toward NH_3_ is higher than 96% under all tested potentials (−0.19, −0.09, and 0.01 V). We also investigated the NITRR performance of Co‐NAs in neutral electrolyte (0.5 m K_2_SO_4_ with 0.1 m NO_3_^−^) and the LSV with CA measurements at stepped potentials are plotted in Figure S12, Supporting Information. Like in KOH, the Co‐NAs show significantly enhanced NITRR activity comparing with Co(OH)_2_‐NAs and the HER LSV shows no obvious onset until −0.6 V versus RHE (Figure 4c). Production of NH_3_ is further quantified and the FE above 96% is obtained from −0.3 to −0.6 V versus RHE (Figure 4d). These results collectively evidence that Co‐NAs can efficiently catalyze NH_3_ production from NITRR under wide application conditions.

**Figure 5:**
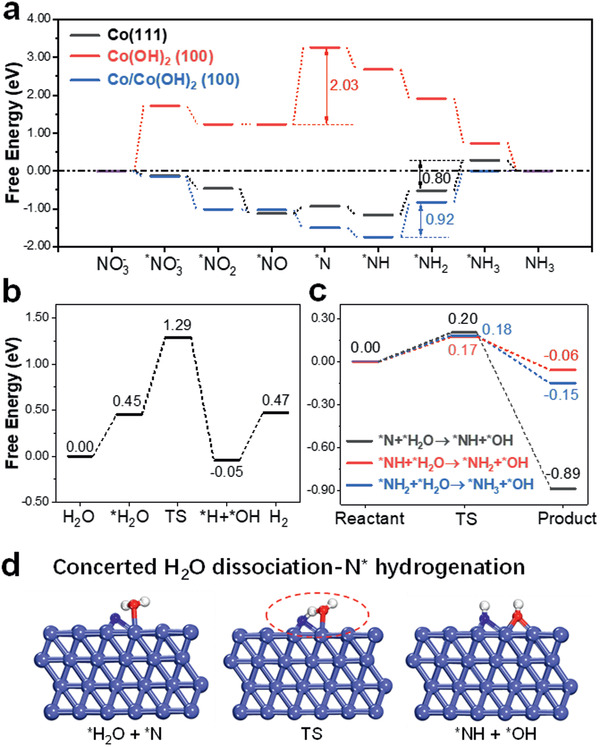
DFT calculations. a) Reaction free energies for different intermediates on Co (111), Co(OH)_2_ (100), and Co/Co(OH)_2_ (100) surfaces toward NITRR. b) Reaction free energies for intermediates on Co (111) toward HER. c) Reaction free energies by considering the concerted H_2_O dissociation and hydrogenation of *N, *NH, and *NH_2_. d) Proposed initial state, transition state, and final state for *N + *H_2_O → *NH + *OH on Co (111) surface. The light blue, blue, red, and white spheres represent Co, N, O, and H atoms, respectively.

To reveal the structure‐activity relationship and further rationalize the superior NITRR activity of Co‐NAs, density functional theory calculations are performed. Co (111) and Co(OH)_2_ (100) surfaces as suggested by the HRTEM investigations are chosen as models. The 8‐electron nitrate reduction reaction is simulated following the gradual deoxygenation of *NO_3_ to form *NO_2_, *NO, *N, and hydrogenation of *N to form *NH, *NH_2_, and *NH_3_ as stepwise intermediates.^[^ ^10^, ^12^ ^]^ The most stable adsorption configurations of above‐mentioned intermediates on Co (111) and Co(OH)_2_ (100) surfaces are presented in Figures S12 and S13, Supporting Information. As plotted in the free energy diagram (**Figure** **5**a), the rate‐determining step (RDS) on Co(OH)_2_ (100) is *NO → *N with an uphill free energy change of 2.03 eV. In contrast, the RDS is calculated to be the hydrogenation of *NH_2_ to *NH_3_ on Co (111) surface with a significantly lower energy change of 0.80 eV, which explains the dramatically‐enhanced NITRR activity of Co‐NAs over Co(OH)_2_‐NAs in our study. Moreover, the free energy change on a Co(OH)_2_‐supported Co surface is calculated to investigate the potential effect of Co^(II)^/Co^(0)^ interface on the NITRR activity (Figure S14, Supporting Information). As can be observed, the RDS of *NH → *NH_2_ with a slightly higher energy change of 0.92 eV is obtained, suggesting the minimal role of Co(OH)_2_/Co heterostructure that could result from the partial reduction of Co(OH)_2_. This is also in good agreement with experimental results presented in Figure S6, Supporting Information, that partially reduced Co(OH)_2_‐NAs exhibit relatively poor NITRR performance.

As aforementioned, one crucial aspect in ammonia production from NITRR is the water‐dissociation reaction (H_2_O → H_2_O → *H + *OH) as it provides a vast number of protons for multiple hydrogenation steps. Therefore, HER process is further simulated on Co (111) surface (Figure S15, Supporting Information).^[^ ^22^ ^]^ As shown in Figure 4b, the Volmer step (H_2_O → *H + *OH), as calculated to be the RDS, exhibits an overall energy change of 1.29 eV over that of Tafel step (2*H → H_2_, 0.52 eV). Such high energy barrier clearly reflects the sluggish water dissociation kinetics and can be well correlated with the poor HER activity. However, it cannot justify the exceptional NH_3_ producing capability at potentials more positive than HER onset (−0.1 V vs RHE) if the water dissociation on Co (111) surface is so severely hindered and the hydrogenation occurs following the common hydrogen‐shuttling model, in which the surface‐adsorbed *H is involved.^[^ ^11^, ^23^ ^]^ To addresses the discrepancies, attention is shifted to the hydrogenation steps (*N → *NH → *NH_2_ → *NH_3_) where the intermediates with unsaturated N atoms are exposed. It is cautiously speculated that *N, *NH and *NH_2_ could functionalize as nucleophiles and are directly participating in the deprotonation (dissociation) of adsorbed H_2_O molecules. To gain theoretical insights, the change of free energy by considering the reactions which involve the simultaenous water dissociation and N(H)‐hydrogenation is presented in Figure 5c (reaction models shown in Figures S16–S18, Supporting Information). Adsorbed *H_2_O and *OH are used as reactants and products in this case. As shown, the uphill energy change of mere 0.18 eV is required for *NH_2_ + *H_2_O → *NH_3_ + *OH (Figure 5d) and this value is significantly lower than that required for the water dissociation step on Co (111). Based on the experimental and theoretical evidence, it is therefore proposed that through the transition states which involves significantly lower energy barrier, the deprotonation of *H_2_O and hydrogenation of *N, *NH, and *NH_2_ on Co (111) surface occur concurrently. This way the kinetically‐sluggish Volmer step is omitted and the sufficient quantity of protons can be supplied from H_2_O molecules at potentials more positive than that required for HER.

## 3 Conclusion

To summarize, Co‐NAs as prepared from the facile electrochemical reduction of Co(OH)_2_ exhibit superior activity and Faradaic efficiency toward NH_3_ production from electrochemical reduction of nitrates. The reduction currents reach −1 and −2.2 A cm^−2^ at −0.16 and −0.24 V versus RHE under alkaline conditions and the NH_3_ FE is above 96% over wide application range. Moreover, the performance is maintained over 10 h under constant current electrolysis at −0.5 A cm^−2^. DFT calculations reveal that metallic Co exhibits optimized intermediates adsorption energy comparing with pristine Co(OH)_2_. Furthermore, it is proposed that the water dissociation process, which provides protons for the hydrogenation steps, is significantly facilitated by the interaction between surface adsorbed *H_2_O and nucleophilic *N, *NH, and *NH_2_ species. This work will guide the design toward efficient NITRR electrocatalysts and the concerted water dissociation–hydrogenation mechanism may open up new opportunities in other catalytic systems.

## 4 Experimental Section

##### Preparation of Co Nanoarrays

Co(OH)_2_ nanoarrays as the precursor were prepared by electrodeposition from aqueous solution of cobalt nitrate hexahydrate (0.05 m). Carbon cloth (CC, 1 cm × 1 cm) was used as the conductive substrate and −1.0 V versus saturated calomel electrode (SCE, without IR‐compensation) was applied for 600 s. The loaded electrode was then rinsed with deionized (DI) water and dried at room temperature. The mass loading of Co(OH)_2_ on CC was measured to be 1.6 mg cm^−2^.To convert the Co(OH)_2_‐NAs to Co‐NAs, the electrodeposited Co(OH)_2_‐NAs were electrochemically reduced at −1.4 V versus Hg/HgO (without IR‐compensation) for 300 s in 1 m KOH electrolyte.

##### General Characterization

The morphology characterization of the electrocatalysts was performed on field‐emission scanning electron microscope (JEM‐F200) and TEM (JEM‐3200FS). XRD were measured with a Bruker D8 Advance instrument (Cu K*α*, 50kV and 360 mA). Spectra of XPS were collected with PHI5000 VersaProbe. The XPS peak positions were calibrated based on C 1s spectrum with the BE of 284.8 eV. UV–vis absorbance spectra were measured on Shimazu UV‐2700 spectrophotometer. The ^1^H NMR signal was recorded on a Bruker 600 MHz system.

##### Electrochemical Measurements

All electrochemical measurements reported in this study were conducted on a Solartron SI1287 potentiostat. The electrocatalysts‐loaded CC (0.5 cm^2^ × 0.5 cm^2^) was used as working electrode. Pt sheet with the size of 1 cm^2^ × 1 cm^2^ was used as counter electrode. Hg/HgO reference electrode was used in 1 m KOH and saturated SCE was used in 0.5 m K_2_SO_4_, respectively. 1 m KOH/0.5 m K_2_SO_4_ with various concentration of potassium nitrate (KNO_3_) was used as the electrolyte for NITRR measurements under alkaline/neutral conditions. All electrochemical measurements were performed in a H‐type two‐compartment cell separated by Nafion 117 membrane. Polarization curves were collected with a scan rate of 5 mV s^−1^ and the electrolyte (60 mL) was stirred at 400 rpm. Automatic IR compensation by current interrupt mode (offtime 27 us, on/off ratio 255) was conducted. The controlled‐potential chronoamperometric (CA) measurements were conducted at stepped potentials for 200 s. The stability of Co‐NAs toward NITRR was evaluated by constant‐current electrolysis (250 and 500 mA cm^−2^) in 80 mL of electrolyte and the potential was monitored for a period of 10 h. The double layer capacitance of the electrocatalysts was measured from −0.70 to −0.60 V for Co(OH)_2_‐NAs and −1.02 to −0.92 V for Co‐NAs with scan rates of 10, 20, 50, 100, 200 mV s^−1^.

##### Quantification of Products and Calculation of Faradaic Efficiency, NH_3_ Production Rate, and Turnover Frequency

For the quantification of NH_3_, the NITRR was conducted in a sealed cell. The quantification was conducted with Nessler's reagent as the coloring agent. Certain amount of electrolyte after NITRR was first taken out from the cathodic compartment and diluted to 5 mL to the linear range of the detection method (0.5–2 ppm NH_4_^+^). Then potassium sodium tartrate solution (500 g L^−1^, 0.1 mL) was added and thoroughly mixed. In the last step 0.1 mL of Nessler's reagent was added to the above mixture. After being left standing for 20 min, the absorbance at 420 nm was measured by UV‐spectroscopy. The obtained value was then fitted to the calibration curve to acquire the corresponding NH_4_^+^ concentration.

NMR was employed to confirm the amount of produced ammonia. For the measurements, known amounts of maleic acid (MA) was employed as internal standard for the quantification of NH_4_^+^. In details, 20 mL of electrolyte after NITRR was mixed with 5 mL of 4 m H_2_SO_4_, to which 10 mg of maleic acid was added as the internal standard. Afterward 50 uL of deuterated‐DMSO was added to 0.5 mL of the above solution for NMR measurements.

The total mass of the NH_4_^+^ in the mixture can be then calculated following the equation:

(1) $$4\times \frac{m_{{\mathrm{NH}}_{4}^{+}}}{M_{{\mathrm{NH}}_{4}^{+}}}/2\times \frac{m_{\mathrm{MA}}}{M_{\mathrm{MA}}}=\mathrm{Are}a_{{\mathrm{NH}}_{4}^{+}}/\mathrm{Are}a_{\mathrm{MA}}$$

where $m_{{\mathrm{NH}}_{4}^{+}}$ and *m*_MA_ are the mass of NH_4_^+^ and maleic acid in test solution, $m_{{\mathrm{NH}}_{4}^{+}}$ and *M*_MA_ are the molecular weights of NH_4_^+^ and maleic acid and $\mathrm{Are}a_{{\mathrm{NH}}_{4}^{+}}$ and Area_MA_ are the areas covered by characteristic peaks in ^1^H‐NMR.

Finally, the NH_4_^+^ concentration in the electrolyte can be calculated based on mass of NH_4_^+^ and the volume of electrolyte taken for the NMR test (20 mL).

The calculation of Faradaic efficiency (FE) toward NH_3_ production was based on:

(2) $$\begin{array}{cc} {\mathrm{FE}}_{{\mathrm{NH}}_{3}}\left(\%\right)= & \left(\mathrm{mole}\;\mathrm{of}\;\mathrm{product}\;\mathrm{measured}\times n\times F\right)/\left(\mathrm{total}\;\mathrm{charge}\;\mathrm{passed}\right)\\ & \times 100\% \end{array}$$

where *n* is 8 for NH_3_ formation from nitrate reduction and F is the Faraday constant (96 485 C mol^−1^).

The NH_3_ production rate (mmol h^−1^ cm^−2^) at specific applied potentials is calculated based on:

(3) $$vNH_{3}=\left(j\times \mathrm{FE}\right)/\left(n\times F\right)\times 3600$$

where *j* is the current density (mA cm^−2^).

The TOF is calculated based on the following equation by assuming all the Co atoms on the electrode are catalytically active:

(4) $$\mathrm{TOF}=\left(j\times \mathrm{FE}\right)/\left(n\times F\times 1000\times n_{\mathrm{Co}}\right)$$

where *n*_Co_ is the mole amount of Co on a 1 cm^2^ × 1 cm^2^ electrode.

The quantitative analysis of gaseous products was performed with on‐line gas chromatograph (Fuli 9790Plus) equipped with a flame ionization detector and thermal detector. During the experiment, a steady Ar flow of 20 mL min^−1^ was purged through the cathodic compartment and fed into the GC.

The FE of H_2_ generation was calculated based on the following equation:

(5) $$\begin{array}{c} {\mathrm{FE}}_{H_{2}}\left(\%\right)=Q_{H_{2}}/Q_{\mathrm{total}}\times 100\%=\left(v/60\right)\times \left(y/22400\right)\times 2\times F\times 100\%/j \end{array}$$

where *v* is the Ar flow rate (20 mL min^−1^), *y* is the calculated H_2_ concentration and *j* is the current density (mA cm^−2^).

##### Theoretical Calculation

Density functional theory calculations were carried out with Perdew–Burke–Ernzerhof exchange correlation in the Gaussian and plane waves formalism as in CP2K code.^[^ ^24^ ^]^ The DZVP basis sets combined with Geodcker‐Teter‐Hutter pseudopotentials were employed. 400 Ry was used for the plane wave density cutoff.^[^ ^25^ ^]^ 1, 5, 6, and 17 valence electrons were considered for H, N, O, and Co, respectively. DFT‐D3 correction was employed to enhance the description of van der Waals interaction.^[^ ^26^ ^]^ DFT+U method with the U‐J being 3.0 eV was used for the on‐site coulomb interaction correction of Co 3d states, as previous study suggested.^[^ ^27^ ^]^ The Brillouin Zone was represented by the Г point in calculating the 6 × 6 Co(111), 5 × 4 Co(OH)_2_(100) and 5 × 4‐Co(100)/6 × 3‐Co(OH)_2_(100) surfaces with a vacuum layer of 15 Å. The convergence threshold for the total energy was set as 1 × 10^−6^ Hartree. Geometry was optimized following the Broyden–Fletcher–Goldfarb–Shanno minimization algorithm until the maximum atomic force was lower than 4.5 × 10^−4^ Hartree per Bohr.^[^ ^28^ ^]^ The involved reaction barriers were calculated by using the climbing image nudged elastic band method, in which the convergence criterion of maximum force on each band was less than 2 × 10^−3^ Hartree per Bohr.^[^ ^29^ ^]^

The NO_3_^−^ reduction reactions were simulated as follows:

(6) $${{}^{*}\mathrm{NO}}_{3}^{-}+H_{2}O\left(l\right)+e^{-}\to {}^{*}NO_{2}+2OH^{-}$$

(7) $${}^{*}NO_{2}+H_{2}O\left(l\right)+2e^{-}\to^{*}\mathrm{NO}+2OH^{-}$$

(8) $${}^{*}\mathrm{NO}+H_{2}O\left(l\right)+2e^{-}\to {}^{*}N+2OH^{-}$$

(9) $${}^{*}N+H_{2}O\left(l\right)+e^{-}\to {}^{*}\mathrm{NH}+OH^{-}$$

(10) $${}^{*}\mathrm{NH}+H_{2}O\left(l\right)+e^{-}\to {}^{*}NH_{2}+OH^{-}$$

(11) $${}^{*}NH_{2}+H_{2}O\left(l\right)+e^{-}\to {}^{*}NH_{3}+OH^{-}$$

(12) $${}^{*}NH_{3}\to NH_{3}\left(g\right)+{}^{*}$$

where * represents the adsorption site. The free energies for each reaction are computed as follows:

(13) ΔG=ΔE+ΔZPE−TΔS

where Δ*E* is the difference of electronic energy between products and reactants, ΔZPE is the change of zero‐point energies, and Δ*S* is the entropy change. Only the vibrational modes of adsorbates were considered for the zero‐point energies calculations. The entropies of molecules in the gas phase were taken from the NIST database. While the entropies of adsorbate and adsorption site were negligible as suggested previously in literature.^[^ ^12^ ^]^

## Conflict of Interest

The authors declare no conflict of interest.

## Supporting information

Supporting Information
