# Supplementary material for: Metallic Co Nanoarray Catalyzes Selective NH3 Production from Electrochemical Nitrate Reduction at Current Densities Exceeding 2 A cm−2
Source: Adv Sci (Weinh). 2021 Feb 1;8(7):2004523. doi: 10.1002/advs.202004523 (PMC8025016; doi:10.1002/advs.202004523)
Supplement: Supplementary file 1 — Supporting Information [file ADVS-8-2004523-s001.pdf]

© 2021 Wiley-VCH GmbH

ADVANCED SCIENCE
Open Access

Supporting Information

for *Adv. Sci.,* DOI: 10.1002/advs.202004523

Metallic Co Nanoarray Catalyzes Selective NH3 Production from Electrochemical Nitrate Reduction at Current Densities Exceeding 2 A cm-2

Xiaohui Deng, Yongpeng Yang, Lei Wang, Xian-Zhu Fu and Jing-Li Luo**

Supplementary Information For

Metallic Co Nanoarray Catalyzes Selective NH_3 Production from Electrochemical Nitrate Reduction at Current Densities Exceeding 2 A cm^{-2}

Xiaohui Deng,[a] Yongpeng Yang[b], Lei Wang,[a] Xian-Zhu Fu[a] and Jing-Li Luo*[a]*

[a] Shenzhen Key Laboratory of Polymer Science and Technology, Guangdong Research Center for Interfacial Engineering of Functional Materials, College of Materials Science and Engineering, Shenzhen University, Shenzhen, China

[b] Henan Institute of Advanced Technology,Zhengzhou University,Zhengzhou, China

Results and Discussion

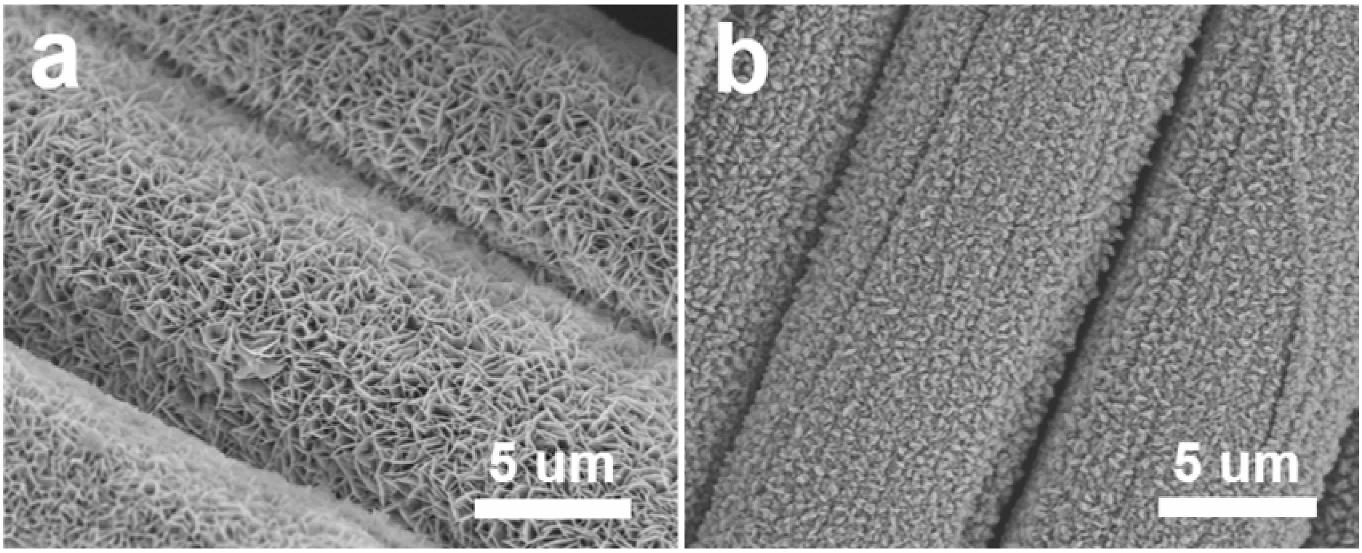

Figure S1. Low-magnification SEM images of $Co(OH)_2$-NAs (a) and Co-NAs (b) on carbon cloth.

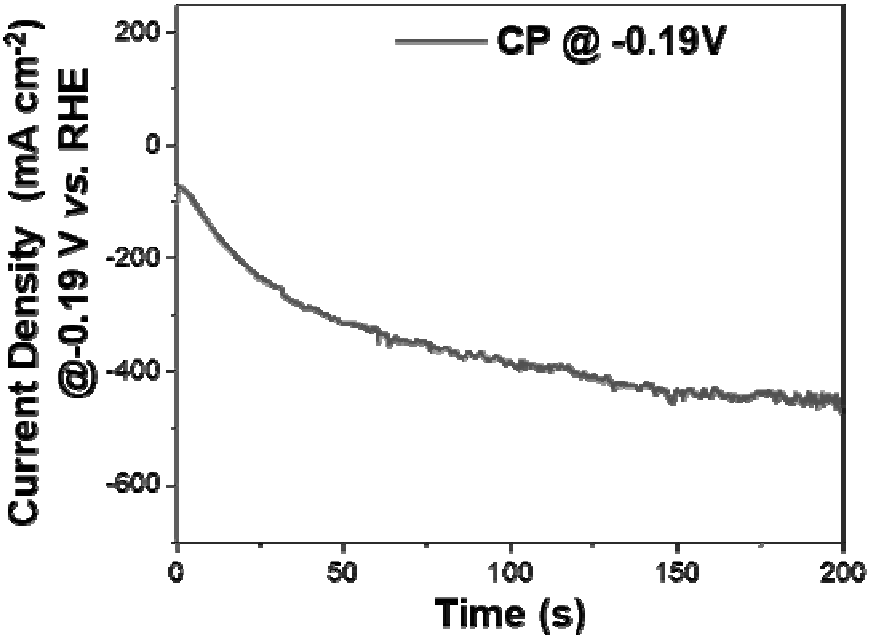

FigureS2. Chronoamperometry measurement of $Co(OH)_2$-NAs catalyzing NITRR at -0.19 V *vs*. RHE.

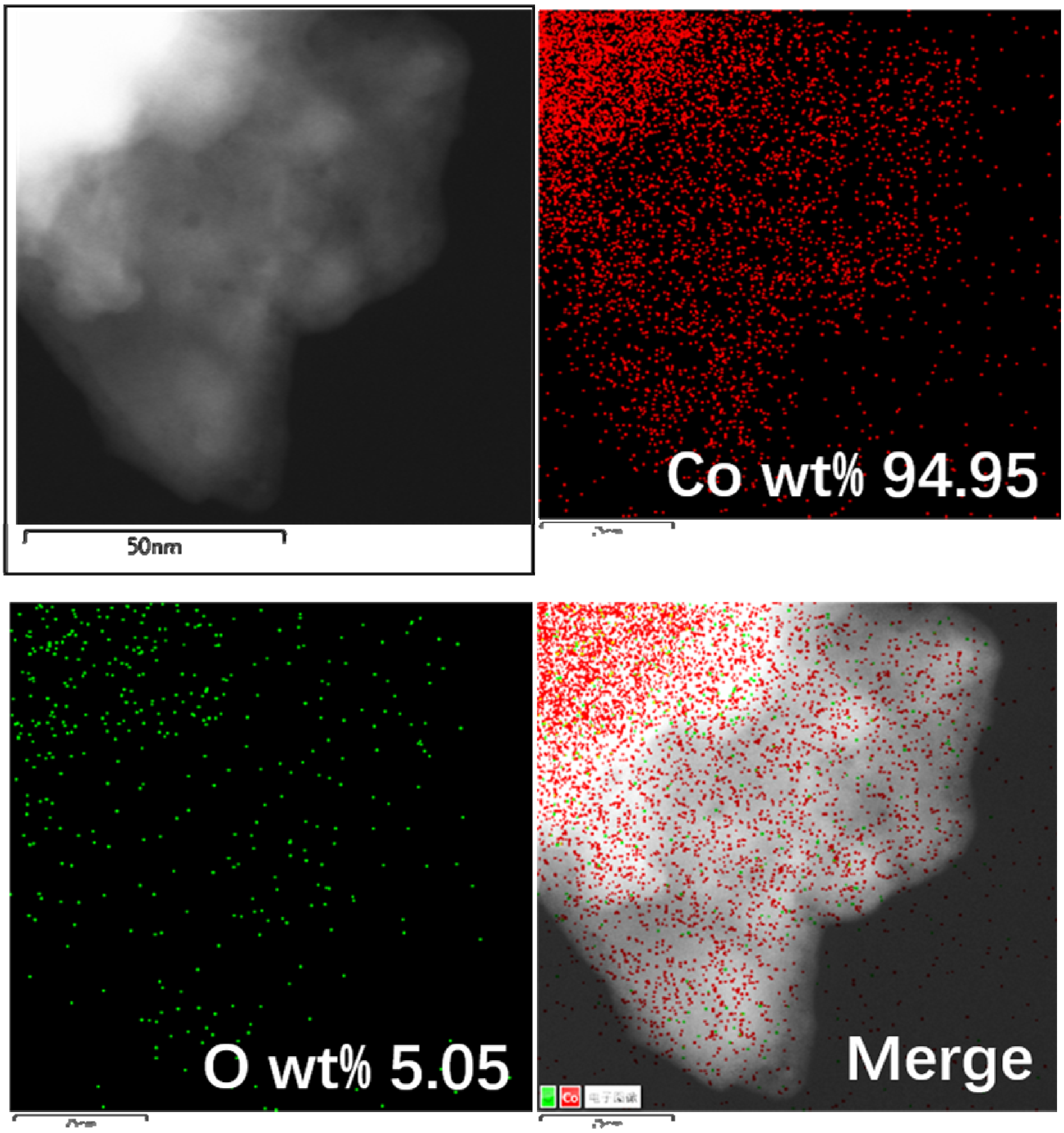

Figure S3. Elemental mapping of Co-NAs.

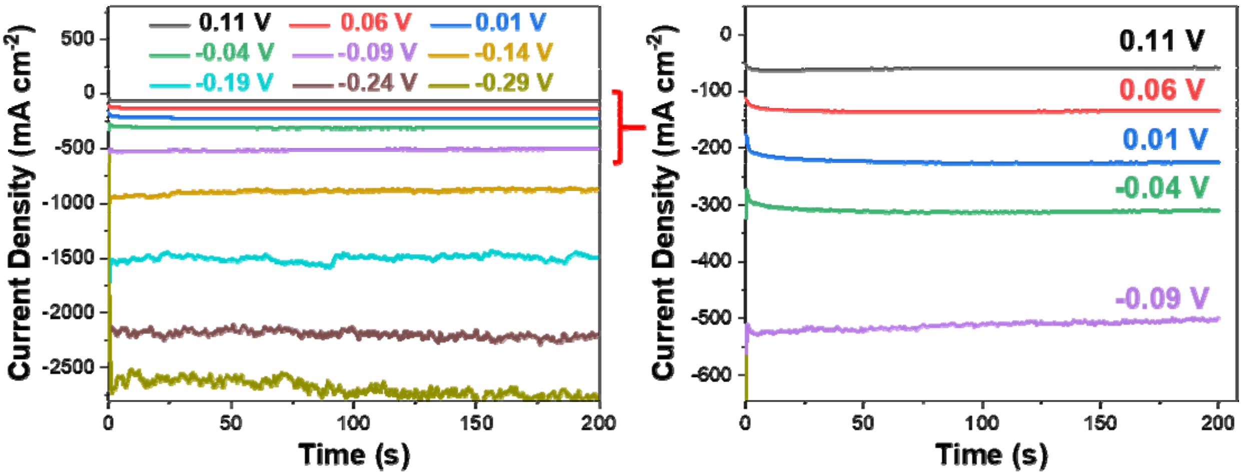

Figure S4. Controlled-potential chronoamperometric (CA) measurementsof Co-NAs catalyzing NITRR. Steady-state currents are collected for 200 s at each potential step.

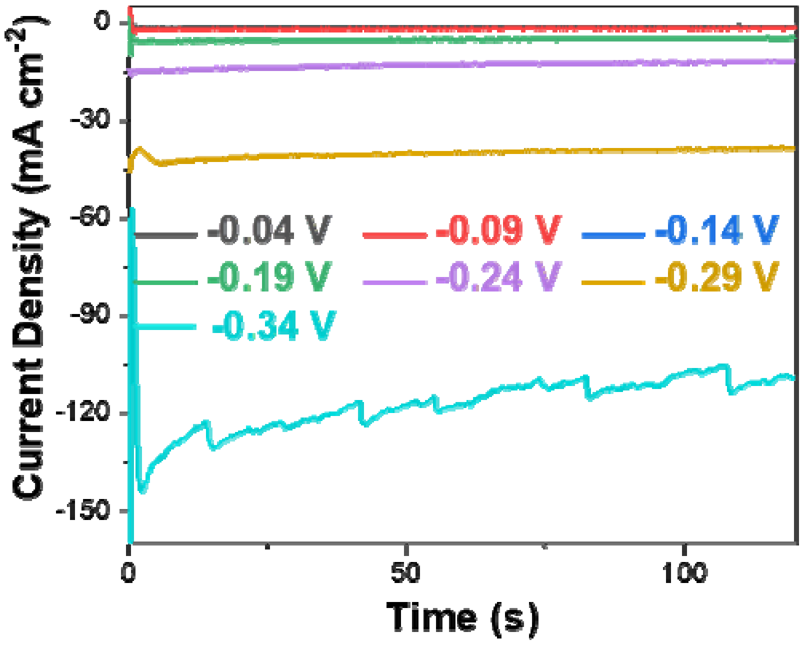

Figure S5. Controlled-potential chronoamperometric (CA) measurements of Co-NAs catalyzing HER. Steady-state currents are collected for 200 s at each potential step.

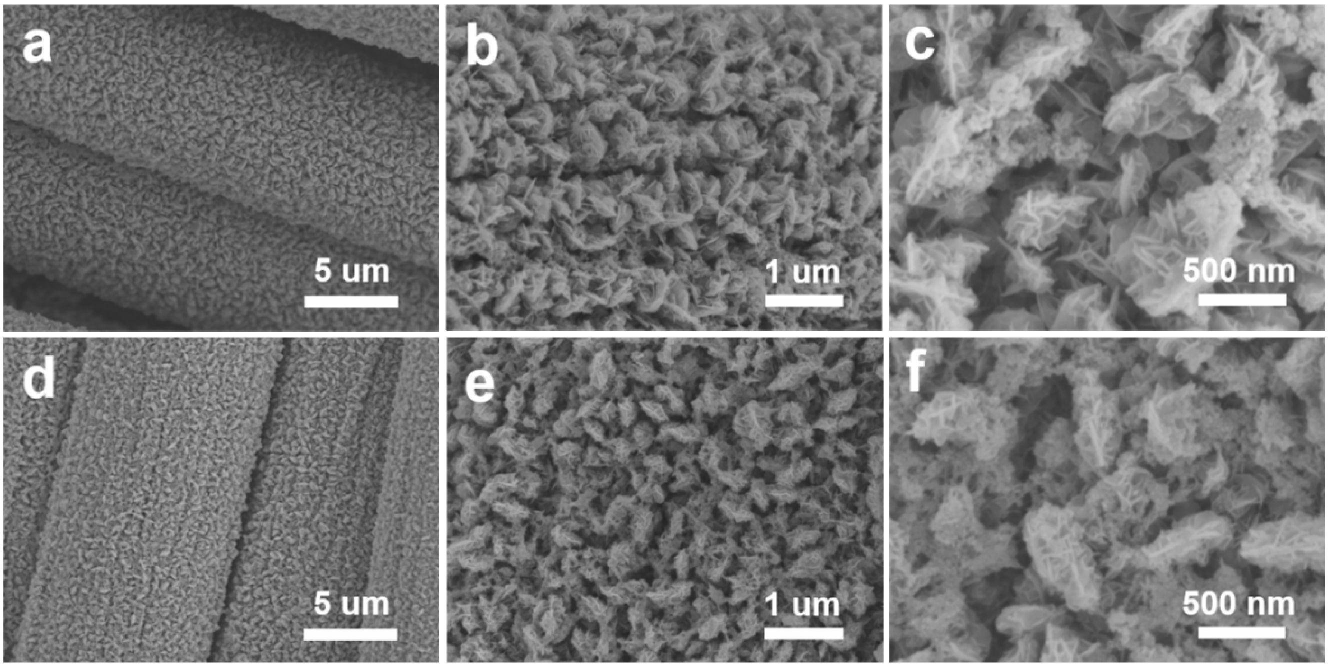

Figure S6. SEM images of $Co(OH)_2$ reduced under various potentials (a-c, -0.29 V *vs.* RHE; d-f. -0.39 V *vs.* RHE).

Figure S7. Chronoamperometric (CA) measurements towards NITRR at -0.19 V *vs.* RHE catalyzed by $Co(OH)_2$-NAs reduced under various potentials for 300 s.

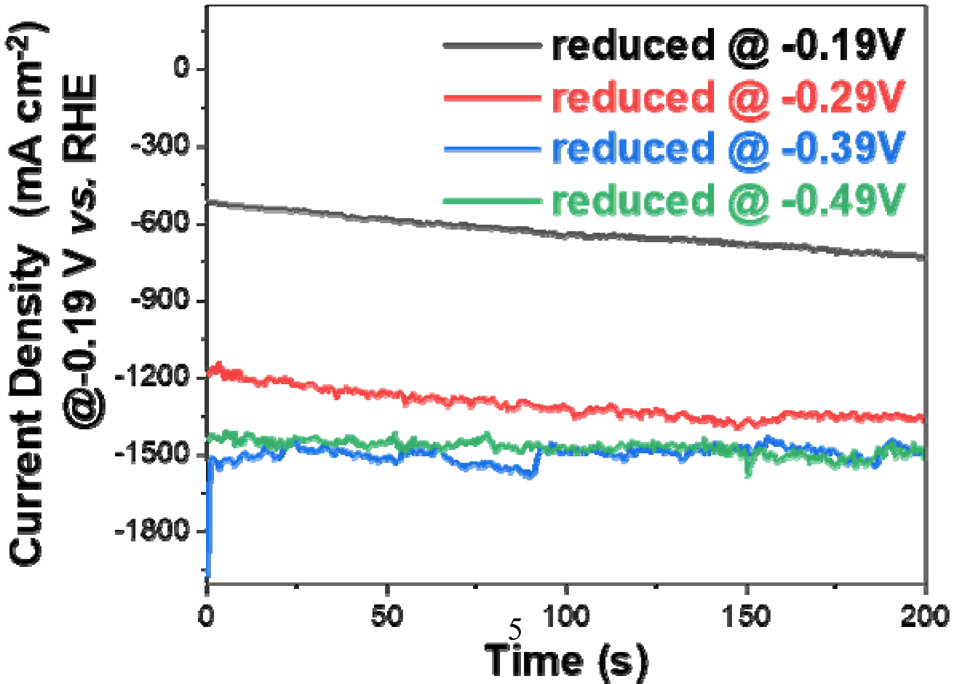

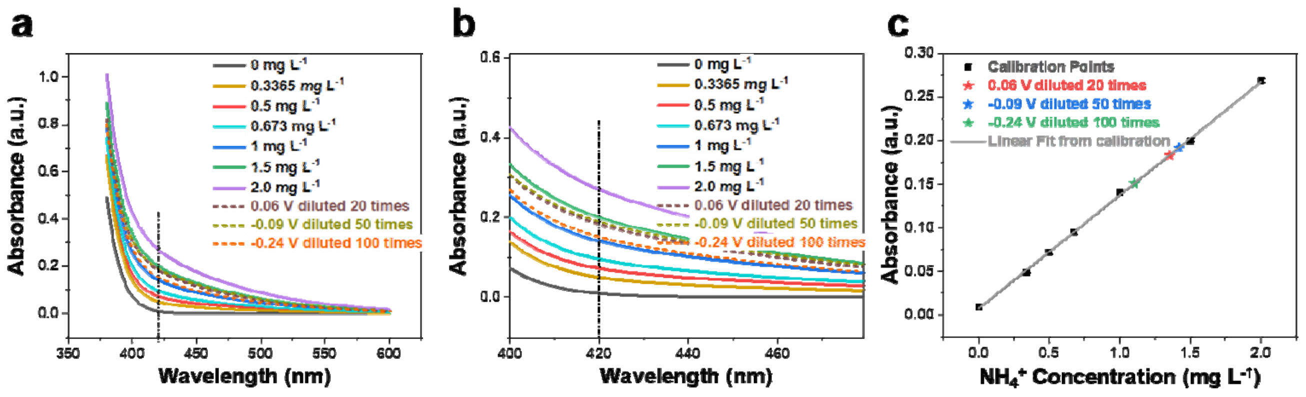

Figure S8.(a, b) The ultraviolet-visible adsorption spectra of standard solutions with different ammonia concentrations and diluted electrolytecollected at 0.06 V, -0.09 V and -0.24 Vas measured by colorimetric method using Nessler's reagent. (c) Obtained concentration-absorbance calibration curve and fitting results of experimental data.

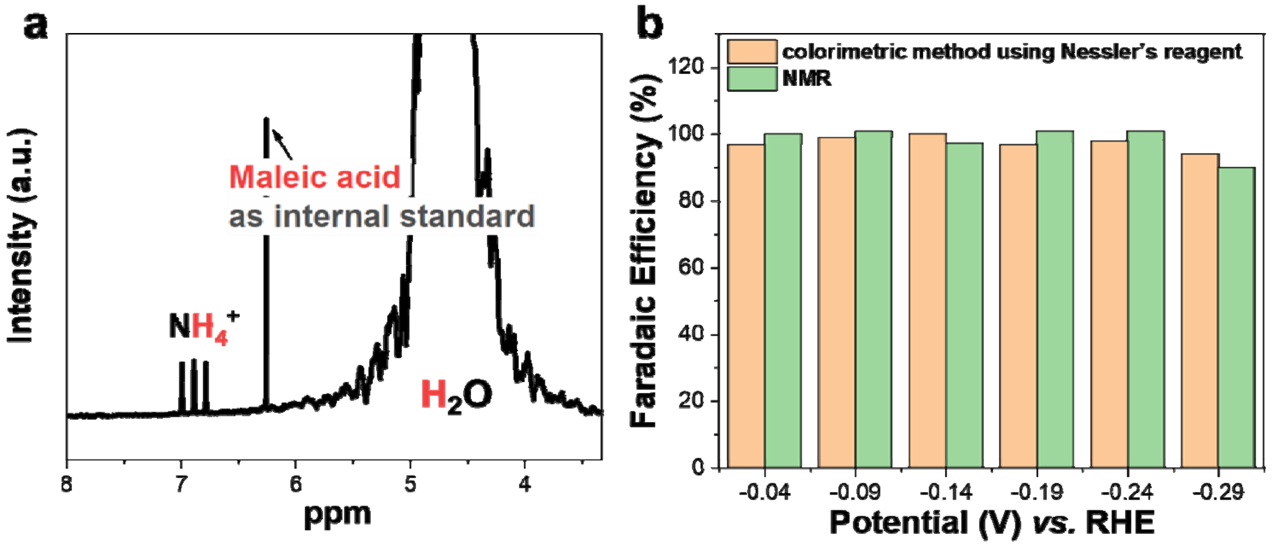

Figure S9. a) ^{1}H-NMR spectrum of the electrolyte after NITRR at -0.24 V. b)Comparison of NH_3 quantification results as measured by colorimetric method (using Nessler's reagent) and NMR method.

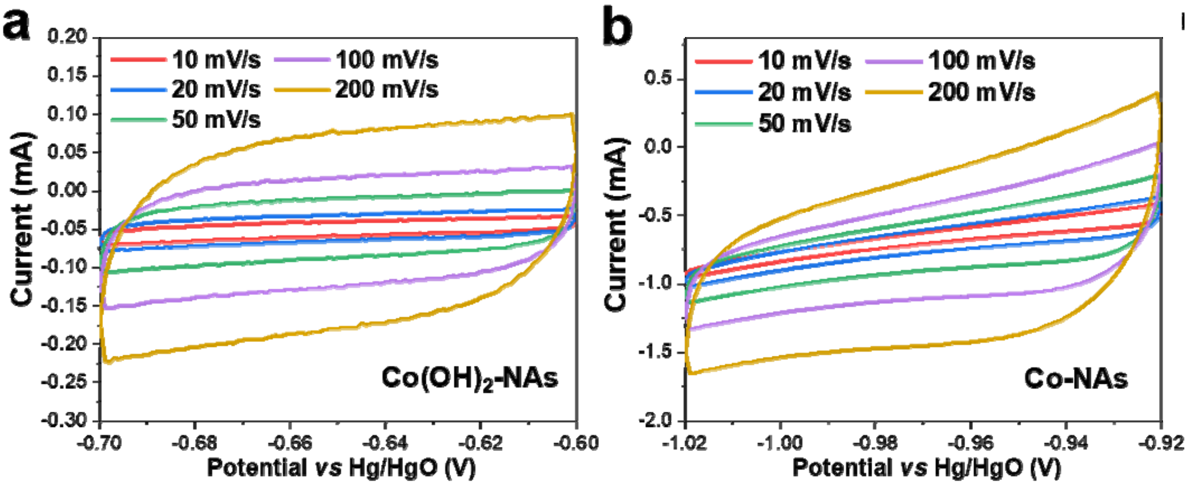

Figure S10.CV curves collected at different scan rates (a, b) of $Co(OH)_2$-NAs and Co-NAs.

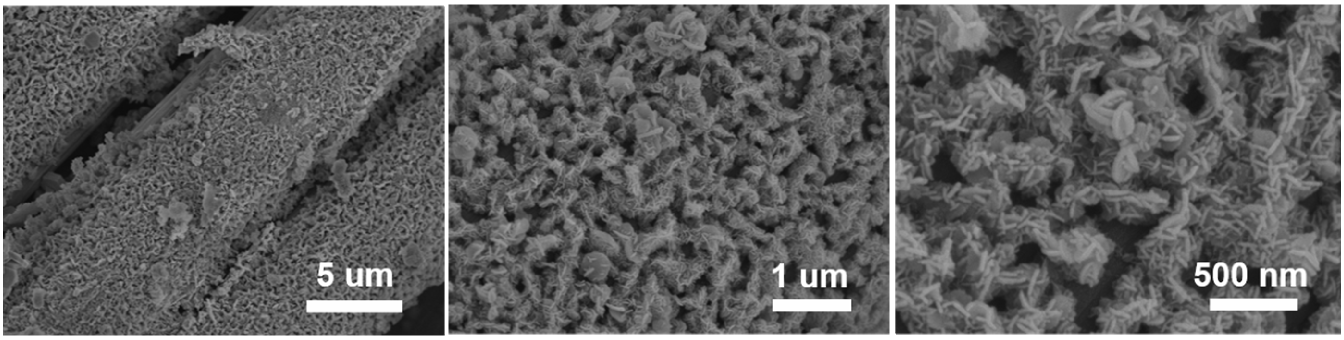

Figure S11. SEM images of Co-NAs after catalyzing long-term NITRR.

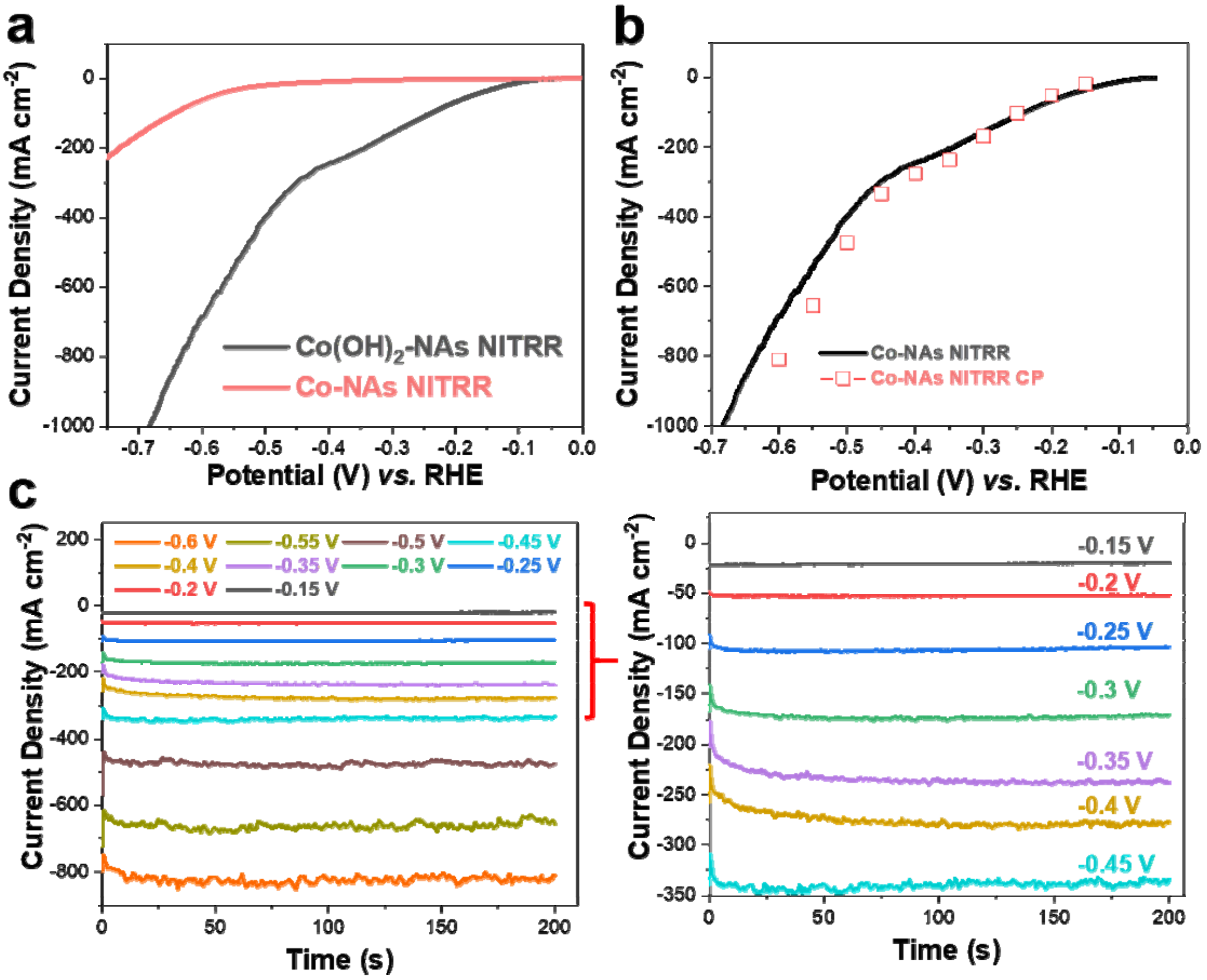

Figure S12. (a) NITRR LSV curves of $Co(OH)_2$-NAs and Co-NAs. (b, c) Chronoamperometric (CA) measurements and steady-state currents collected at stepped potentials during NITRR catalyzed by Co-NAs.

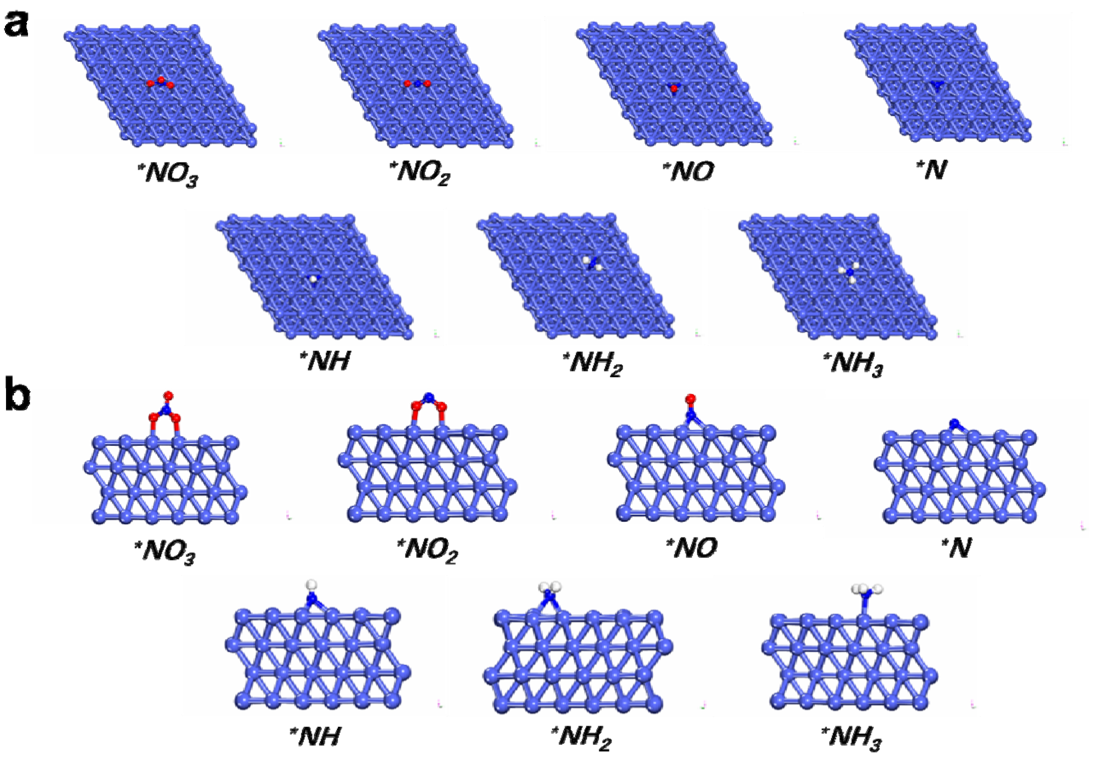

Figure S12. The most stable adsorption configurations of NITRR intermediates on Co (111) surface (a) Top view, (b) Side view.

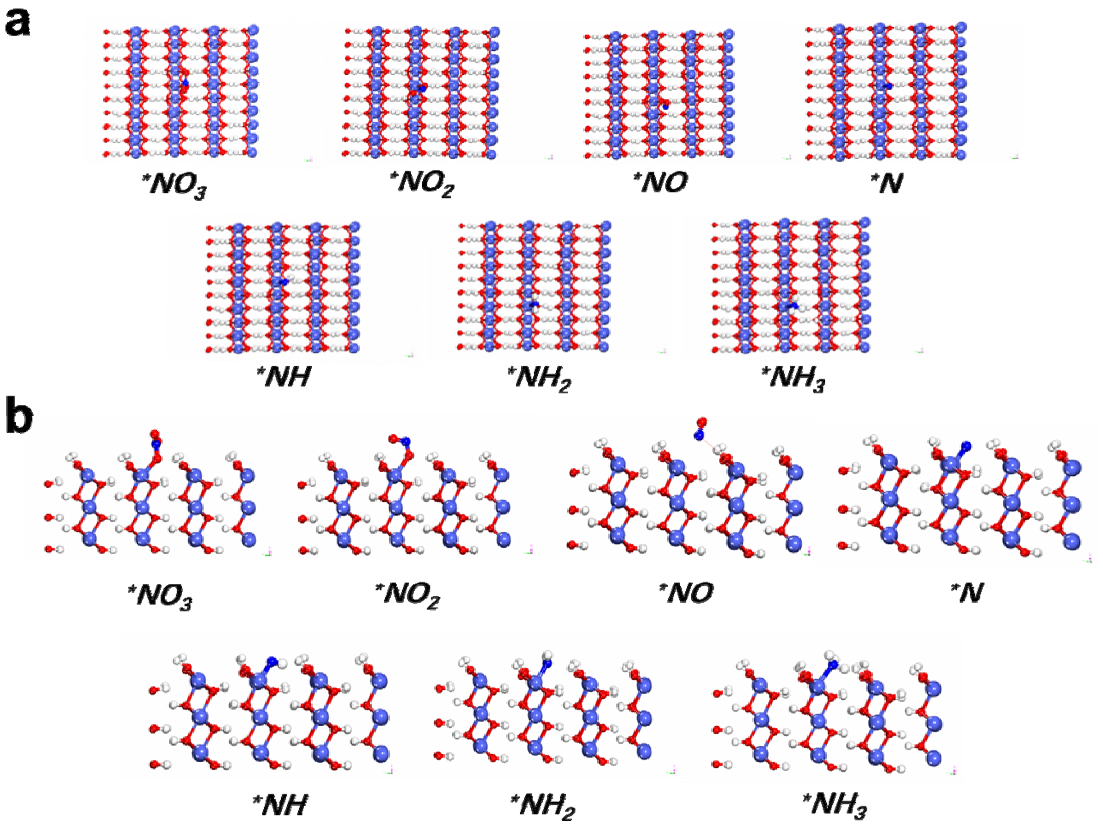

Figure S13. The most stable adsorption configurations of NITRR intermediates on $Co(OH)_2$ (100) surface (a) Top view, (b) Side view.

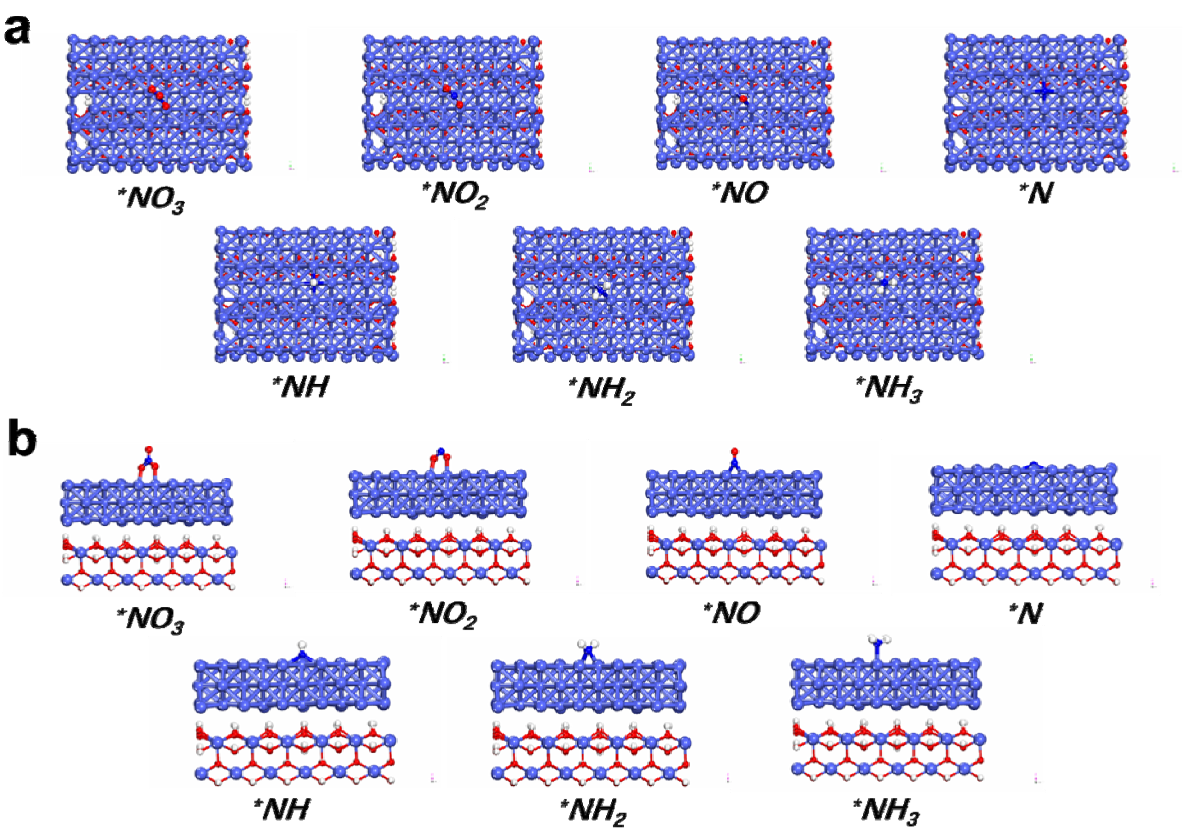

Figure S14. The most stable adsorption configurations of NITRR intermediates on $Co(OH)_2$ (100) surface (a) Top view, (b) Side view.

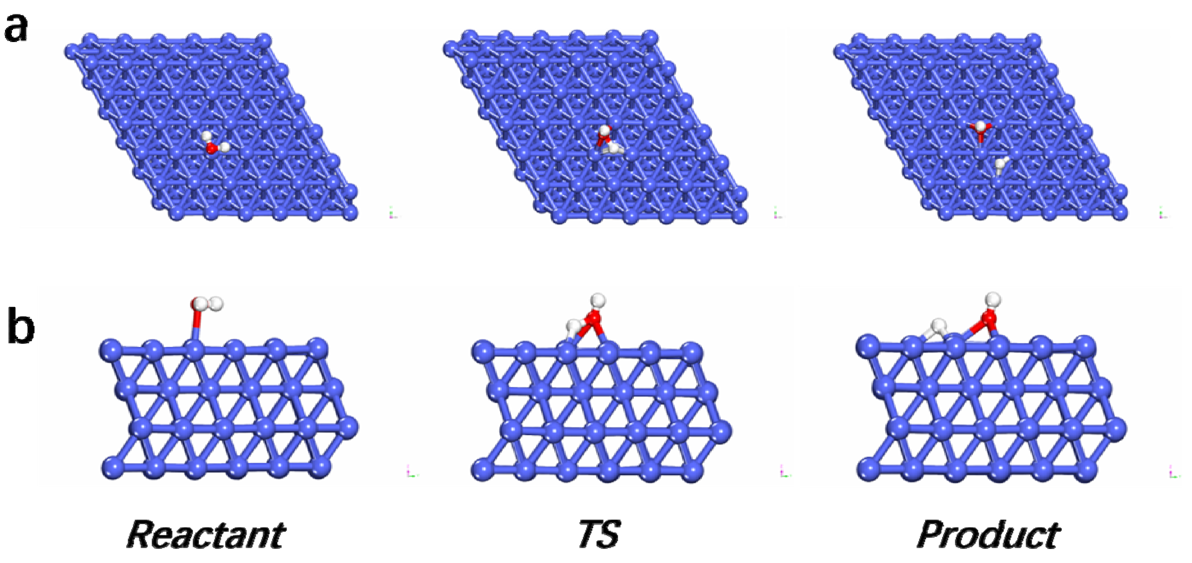

Figure S15. The most stable adsorption configurations of HER intermediates on Co (111) surface during the Volmer process. (a) Top view, (b) Side view.

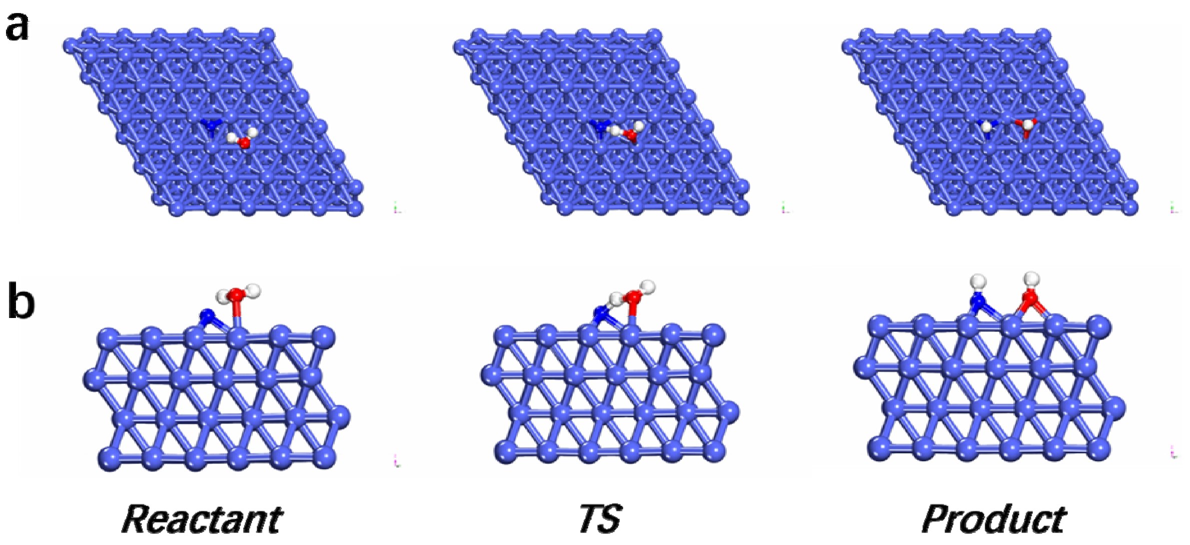

Figure S16. Proposed initial state, transition state and final state for *N + *H_2O → *NH + *OH. (a) Top view, (b) Side view.

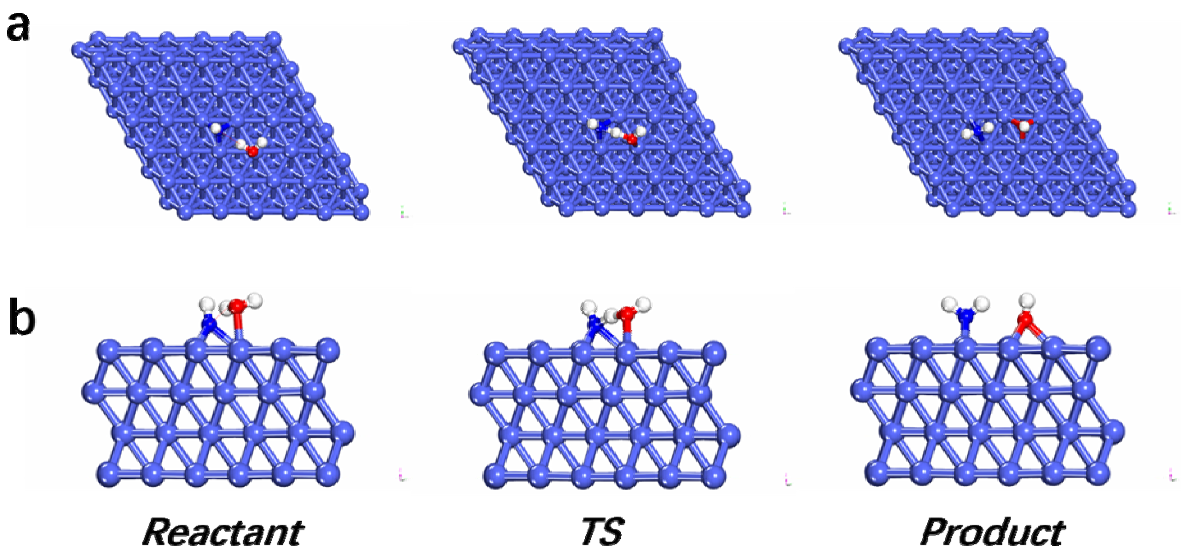

Figure S17. Proposed initial state, transition state and final state for *NH + *H_2O → *NH_2 + *OH. (a) Top view, (b) Side view.

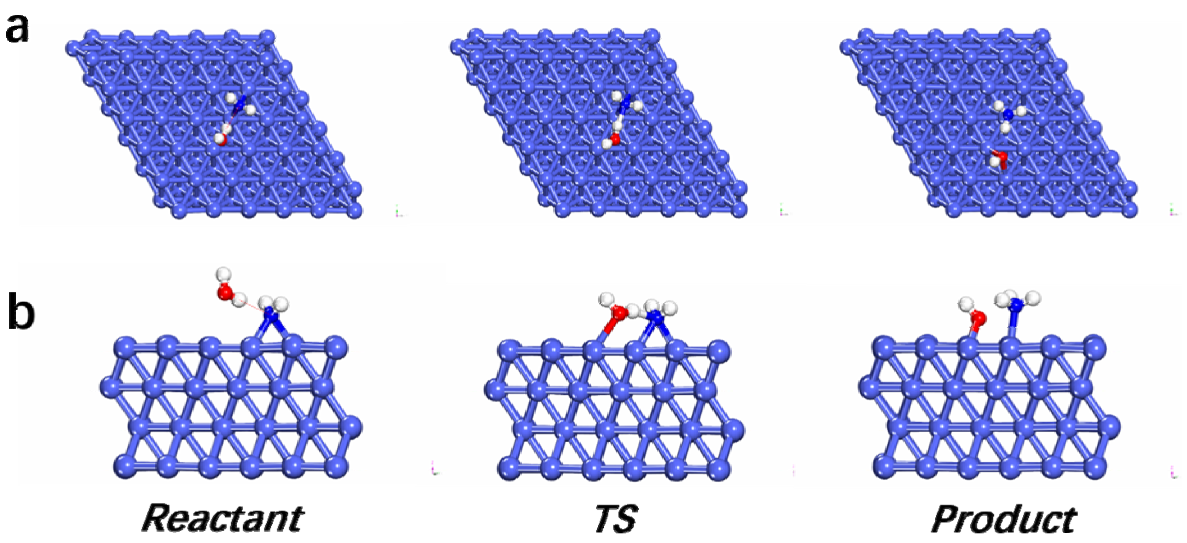

Figure S18. Proposed initial state, transition state and final state for $*NH_2 + *H_2O \rightarrow *NH_3 + *OH$. (a) Top view, (b) Side view.

Table S1. Comparison of NITRR activity and NH_3 production performance between Co-NAs and recently-reported electrocatalysts.

Electrocatalyst	NITRR Conditions	Activity	NH_3 Faradaic Efficiency	NH_3 Production Rate	Reference
Cu-PTCDA	0.1 M PBS<br>500 ppm NO_3^-	~ -15 mA cm^{-2}<br>@ -0.4 V *vs*. RHE	77±3% @ -0.4 V vs. RHE	436±85 ug h^{-1} cm^{-2}	1
O-deficient TiO_2	0.5 M Na_2SO_4<br>50 ppm NO_3^-	-8 mA cm^{-2}<br>@ -1.6 V *vs*. SCE	85% @ -1.6 V *vs*. SCE	0.045 mmol mg^{-1} h^{-1}	2
Cu/Cu_2O nanowire arrays	0.5 M Na_2SO_4<br>200 ppm NO_3^-	-80 mA cm^{-2}<br>@ -0.6 V *vs*. RHE	88 % @ -0.85 V *vs*. RHE	0.24 mmol h^{-1} cm^{-2}	3
Ti	0.1 M HNO_3<br>0.3 M KNO_3	jNH_3 ~ -20 mA cm^{-2}<br>@ -1 V *vs*. RHE	Max FE 82 %	-	4
Au with surface adsorbed thiourea	0.5 M $NaNO_3$<br>pH 12.5	-10 mA cm^{-2}<br>@ -0.5 V *vs*. RHE	85 % @ 2.85 mA cm^{-2}	-	5
Co_3O_4/Ti	1 M KOH<br>50 ppm NO_3^-	-	80 % @ 10 mA cm^{-2}	-	6
$Cu_{50}Ni_{50}$ alloy	1 M KOH<br>0.1 M NO_3^-	-190 mA cm^{-2}<br>@ 0 V *vs*. RHE<br>(400 rpm)	~ 99 % @ -0.2 V *vs*. RHE<br>~ 80 % @ 0 V *vs*. RHE	-	7
Strained Ru nanoclusters	1 M KOH<br>0.1 M NO_3^-	-120 mA cm^{-2}<br>@ -0.2 V *vs*. RHE	100 % @ -0.2 V *vs*. RHE<br>80 % @ 0 V *vs*. RHE	5.56 mol g^{-1} h^{-1}<br>1.17 mmol h^{-1} cm^{-2}<br>@ -0.2 V vs. RHE	8
**Co-NAs**	**1 M KOH<br>0.1 M NO_3^-**	**-100 mA cm^{-2}<br>@ 0.04 V *vs*. RHE<br>-500 mA cm^{-2}<br>@ -0.1 V *vs*. RHE<br>-1000 mA cm^{-2}<br>@ -0.16 V *vs*. RHE<br>-2000 mA cm^{-2}<br>@ -0.23 V *vs*. RHE**	**≥ 97 % from 0.06 to -0.24 V *vs*. RHE**	**2.6 mol g^{-1} h^{-1}/<br>4.16 mmol h^{-1} cm^{-2}<br>@ -0.14 V vs. RHE<br>6.52 mol g^{-1} h^{-1}/<br>10.43 mmol h-^{1} cm^{-2}<br>@ -0.24 V vs. RHE**	**This work**
**Co-NAs**	**0.5 M Na_2SO_4<br>0.1 M NO_3^-**	**-250 mA cm^{-2}<br>@ -0.4 V *vs*. RHE<br>-680 mA cm^{-2}<br>@ -0.6 V *vs*. RHE**	**≥ 96 % from -0.3 to -0.6 V *vs*. RHE**	**1.98 mol g^{-1} h^{-1}/<br>3.17 mmol h-^{1} cm^{-2}<br>@ -0.6 V vs. RHE**	**This work**

References

[1] G.-F. Chen, Y. Yuan, H. Jiang, S.-Y. Ren, L.-X. Ding, L. Ma, T. Wu, J. Lu, H. Wang, *Nat. Energy***2020**, *5*, 605-613.

[2] R. Jia, Y. Wang, C. Wang, Y. Ling, Y. Yu, B. Zhang, *ACS Catal.* **2020**, *10*, 3533-3540.

[3] Y. Wang, W. Zhou, R. Jia, Y. Yu, B. Zhang, *Angew. Chem. Int. Ed.* **2020**, *59*, 5350-5354.

[4] J. M. McEnaney, S. J. Blair, A. C. Nielander, J. A. Schwalbe, D. M. Koshy, M. Cargnello, T. F. Jaramillo, ACS Sustainable Chem. Eng. **2020**, *8*, 2672-2681.

[5] M. S. El-Deab, *Electrochim. Acta***2004**, *49*, 1639-1645.

[6] L. Su, K. Li, H. Zhang, M. Fan, D. Ying, T. Sun, Y. Wang, J. Jia, *Water Res.***2017**, *120*, 1-11.

[7] Y. Wang, A. Xu, Z. Wang, L. Huang, J. Li, F. Li, J. Wicks, M. Luo, D.-H. Nam, C.-S. Tan, Y. Ding, J. Wu, Y. Lum, C.-T. Dinh, D. Sinton, G. Zheng, E. H. Sargent, *J. Am. Chem. Soc.***2020**, *142*, 5702-5708.

[8] J. Li, G. Zhan, J. Yang, F. Quan, C. Mao, Y. Liu, B. Wang, F. Lei, L. Li, A. W. M. Chan, L. Xu, Y. Shi, Y. Du, W. Hao, P. K. Wong, J. Wang, S.-X. Dou, L. Zhang, J. C. Yu, *J. Am. Chem. Soc.* **2020**, *142*, 7036-7046.
